# NIR TADF emitters and OLEDs: challenges, progress, and perspectives

**DOI:** 10.1039/d2sc02201j

**Published:** 2022-07-11

**Authors:** Yuxin Xiao, Hailan Wang, Zongliang Xie, Mingyao Shen, Rongjuan Huang, Yuchen Miao, Guanyu Liu, Tao Yu, Wei Huang

**Affiliations:** Frontiers Science Center for Flexible Electronics (FSCFE), Shaanxi Institute of Flexible Electronics (SIFE) & Shaanxi Institute of Biomedical Materials and Engineering (SIBME), Northwestern Polytechnical University (NPU) Xi'an 710072 China iamtyu@nwpu.edu.cn iamwhuang@nwpu.edu.cn; Key Laboratory of Flexible Electronics of Zhejiang Province, Ningbo Institute of Northwestern Polytechnical University 218 Qingyi Road Ningbo 315103 China; Key Laboratory of Flexible Electronics & Institute of Advanced Materials, Nanjing Tech University 30 South Puzhu Road Nanjing 211816 China; Key Laboratory for Organic Electronics and Information Displays & Institute of Advanced Materials, Nanjing University of Posts and Telecommunications Nanjing 210023 China

## Abstract

Near-infrared (NIR) light-emitting materials show excellent potential applications in the fields of military technology, bioimaging, optical communication, organic light-emitting diodes (OLEDs), *etc.* Recently, thermally activated delayed fluorescence (TADF) emitters have made historic developments in the field of OLEDs. These metal-free materials are more attractive because of efficient reverse intersystem crossing processes which result in promising high efficiencies in OLEDs. However, the development of NIR TADF emitters has progressed at a relatively slower pace which could be ascribed to the difficult promotion of external quantum efficiencies. Thus, increasing attention has been paid to NIR TADF emitters. In this review, the recent progress of NIR TADF emitters has been summarized along with their molecular design strategies and photophysical properties, as well as electroluminescence performance data of their OLEDs, respectively.

## Abstract 2

This review presents the recent progress of NIR TADF emitters along with their molecular design strategies and photophysical properties, as well as the electroluminescence performance data of the emitters and their OLEDs.

## Graphical abstract

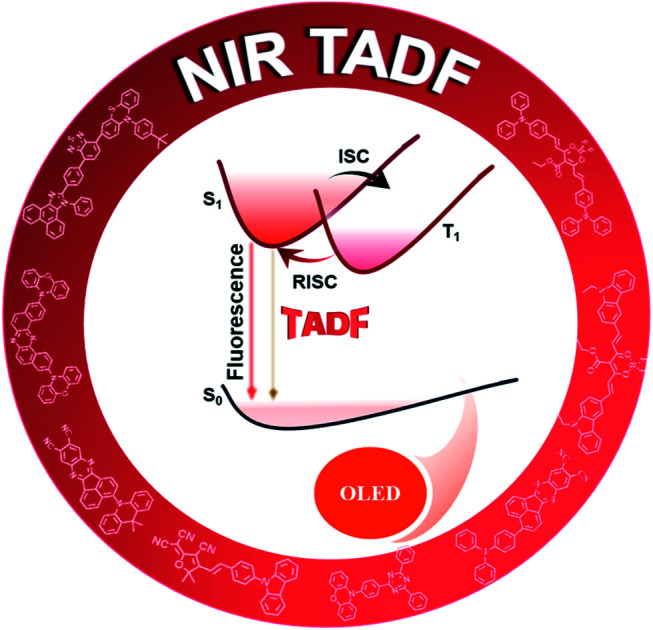

## Introduction

Infrared light is defined as one kind of electromagnetic radiation whose wavelength is between those of visible light and microwaves. Within this range, the near-infrared (NIR) region typically spans from the longest wavelength of red light (680 nm) to 2500 nm.^1^ In recent years, NIR materials have attracted great attention in organic light-emitting diodes (OLEDs),^2,3^ solar cells,^4–6^ bioimaging,^7–10^ information storage,^11,12^ and phototherapy devices.^13^ As one of the most important applications of NIR luminescent materials, NIR OLEDs play important roles not only in the fields of night-vision devices, optical communication and information-secured displays, but also in the fields of flat panel displays and lighting fixtures.^14–18^ Many types of emitters have been adopted in NIR OLEDs, such as metal complexes, conjugated polymers, phosphorescent metal complexes, *etc.*^19^ These materials endow NIR OLEDs with excellent features such as light weight, low power consumption, fast response time, good processing performance, wide temperature range, low cost and so on. However, conventional NIR OLEDs usually suffer from inferior efficiencies due to the low exciton utilization. In recent years, researchers have successfully developed NIR thermally activated delayed fluorescence (NIR TADF) materials, which are able to make full use of excitons and theoretically achieve 100% internal quantum efficiency (IQE). Therefore, the research on NIR TADF materials has become a hot topic nowadays.

**Fig. 1:**
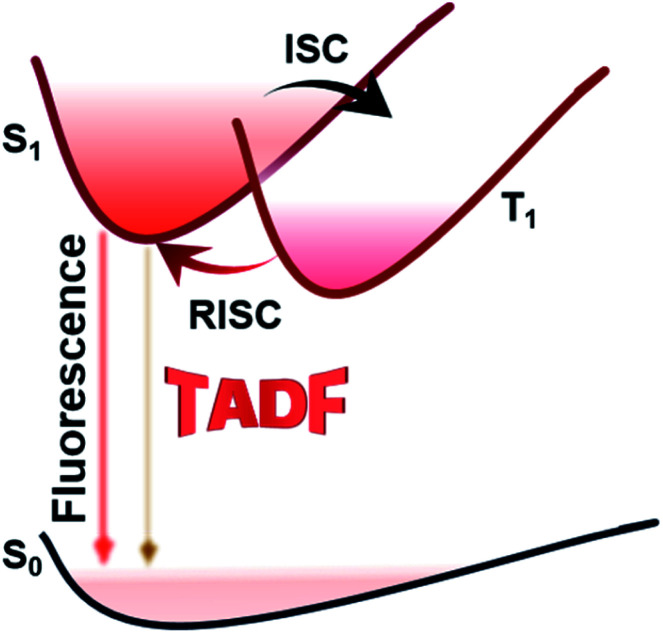
Schematic diagram of the TADF mechanism.

TADF is not a new concept. It was first reported in 1929 by Perrin *et al.*^20^ Since then, TADF emitters have been made commercially available by many industrial companies, such as Kyulux and CYNORA.^21^ Chihaya Adachi utilized the TADF mechanism to build an efficient OLED in 2012.^22^ Unlike traditional fluorescent and phosphorescent materials, TADF emitters often have a narrow enough energy split (Δ*E*_ST_) between the excited singlet (S_1_) and triplet (T_1_) states to allow reverse intersystem crossing (RISC) processes at ambient temperature (Fig. 1). Thus, remarkable high external quantum efficiencies (EQEs) which are comparable to those of advanced phosphorescent emitters could also be realized.^23^ However, designing NIR delayed fluorescence materials is more difficult compared with designing other TADF emitters in the visible region due to the requirement of a relatively small energy gap.

In this review, we summarize the recent progress of NIR TADF emitters and their applications in OLEDs. The molecular design strategy of NIR TADF emitters, the relationship between molecular structures and photophysical properties, and device performances are comprehensively discussed in this review. The photophysical properties of these TADF materials are summarized in Table 1 and the electroluminescence (EL) characteristics of the corresponding OLEDs are listed in Table 2. Moreover, the prospects of the TADF molecular design strategy are discussed to optimize the photophysical properties of TADF emitters and the performances of OLED devices. We believe that this review will have a beneficial impact on the design of a variety of NIR TADF emitters and OLEDs, which will arouse more research interest in this field.

**Table 1:** Summary of the photophysical and electrochemical properties of TADF emitters discussed in this review

Emitter	*E*_S_/*E*_T_ [eV]	Δ*E*_ST_ [eV]	Solid state *λ*_PLmax_/PLQY/*τ*^a^ [nm/%/μs]	Solution state *λ*_PLmax_/PLQY/*τ*^a^ [nm/%/μs]	HOMO/LUMO [eV]	Ref.
TPA-DCPP	2.38/2.25	0.13	708^b^/14^b^/0.76^b^	588/84/—	−5.30/−3.52	30
POZ-DBPHZ	2.28/2.26	0.02^d^	595^d^/79^d^/—	521/0.33/—	−5.36/−3.38	31
APDC-DTPA	2.16/2.02	0.14	756/17/—	639/—/—	−5.27/−3.45	32
DPA-Ph-DBPzDCN	2.34/2.11	0.23	765^b^/2^b^/—	618/91/—	−5.26/−3.63	33
TPA-PZCN	2.12/1.99	0.13	623/—/—	585/—/—	−5.00/−2.73	34
HANTA-tCz	2.17/2.05	0.12^e^	650^d^/50^d^/5.81^d^	637/—/9.90	−5.54/−3.65	35
HANTA-tPCz	2.16/2.05	0.11^e^	655^d^/38^d^/4.94^d^	660/—/4.70	−5.38/−3.68	35
DCPPr-TPA	2.03/1.79	0.24	702^b^/11^b^/50.5^b^	606/83/—	−5.31/−3.65	36
DCPPr-β-NDPA	1.97/1.78	0.19	710^b^/7^b^/28.2^d^	612/74/—	−5.31/−3.65	36
DCPPr-α-NDPA	2.06/1.86	0.23	692^b^/13^b^/42.7^d^	598/82/—	−5.25/−3.64	36
DCPPr-NBPPA	1.99/1.76	0.23	702^b^/6^b^/35.6^d^	620/72/—	−5.27/−3.65	36
TPA-PZTCN	1.64/1.50^c^	0.14	729^d^/21.7^d^/18.6^d^	674/77.7/—	−5.66/−3.84	37
*p*CNQ-TPA	1.98/1.80	0.18	724/31.4/13.2	624/94.3/11.7	−5.78/−4.02	38
CNPP-TPA	2.30/2.22	0.08^f^	706^d^, 748^b^/81.1^d^, 15.1^b^/8.07^d^, 5.18^b^	631/81.4/	−5.76/−4.11	39
AQTC-DTPA	2.06/1.88	0.18	718^d^, 878^b^/19.10^d^, 1.10^b^/7.95^d^, 9.70^b^	636/44.10/4.07	−5.19/−3.11	40
TPA-QCN	2.13/2.04^c^	0.23	733^b^/21^b^/0.8^b^	583/—/—	−5.22/−3.48	41
TPA-PPDCN	2.18/2.02^c^	0.23	725^b^/0.21^b^/1.96^b^	613/—/—	−5.24/−3.22	42
TPAAP	2.28/2.09	0.19^c^	777^b^/20.3^b^/10.4^b^	609/97.3/—	−5.38/−3.39	43
CAT-1	1.45/1.34	0.11	763, 950^b^/8.8, <0.18^b^/—	770/3.9/	−5.64/−4.11	44
TPAAQ	2.46/2.13	0.33^c^	716^b^/16.3^b^/8.12^b^	560/93.0/—	−5.38/−3.26	43
mDPBPZ-PXZ	2.29/2.25	0.04^d^	*ca.* 610/95 ± 1.3^d^/7.4	638/—/—	−5.39/−3.32	45
DCPA-TPA	—	—	824^b^, 750^d^/—/8, 33	656/—/—	−5.31/−3.62	46
DCPA-BBPA	—	—	854^b^, 783^d^/—/3, 16	672/—/—	−5.23/−3.61	46
TPAAZ	2.04/1.97	0.07	1009^b^/—/—	742/7.7/—	−5.3/−4.1	47
dpTPAAP	2.22/1.97	0.25	760^d^, 802^b^/38.5^d^, 13.0^b^/—	624/97.4/—	−5.31/−3.42	48
dpTPAAZ	1.95/—	—	764^d^/5.9^d^/5.6^d^	763/7.6/—	−5.32/−3.94	48
SDPA-APDC	2.23/2.12	0.11	758^b^/15^b^/—	631/—/—	−5.19/−3.39	49
DA-*p*CNPPZ	1.71/1.58	0.13	—	730/20/11.2	—	50
CN-TPA	2.15/2.05	0.10	—/98/5.77	675/—/—	−5.11/−3.09	51
TPAAP-D	2.07/1.94	0.13	—	597/98.2/—	−5.26/−3.05	52
TPAAQ-D	2.25/1.99	0.26	—	552/94.6/—	−5.27/−2.88	52
CAT-TPE	—	0.08^g^	752^d^/11.3^d^/—	727/—/—	−5.51/−3.94	53
TPA–CN–N4	2.34/2.14	0.20	—	632/93/—	−5.13/−2.94	54
TPA–CN–N4-2PY	2.22/2.08	0.14	—	653/68/—	−5.12/−3.01	54
MPPA-MCBP	—	*ca.* 0.17	690/—/0.67	667/—/—	−5.11/—	55
TPA-cNDI	—	—	736/0.028/—	—	—	56
BDC-1	2.0/1.94	0.06	782/0.035/—	795/0.001/—	−5.5/−3.8	57
CAZ-A	2.27/2.07(T_2_)	0.20	698 (neat)/0.02/—	—	—	58
BDC-2	2.01/1.69	0.32	801/0.041/—	788/0.013/—	−5.6/−3.7	59
TPAM-BF_2_			764^d^/—/13.7^d^		−5.35/−3.7	60
CzTCF	2.05/1.95	0.10	675/16.5/—	590/8.57/—	−6.11/−4.31	61
tBCzTCF	1.88/1.79	0.09	712/26.6/—	619/11.2/—	−5.95/−4.35	61
PIBz-3-PTZ	2.39/2.35	0.04	643^b^/35^b^/—	580/—	−4.97/−3.16	63
TPACNBz	2.03/1.97	0.06	750^b^, 710^d^/21^b^, 52^d^/—	674/70/6	−5.60/−3.70	64
R-TBN	1.79/1.63	0.16	698^d^/0.31^d^/—	692/46.4/100	−4.69/−3.00	68
R-DOBP	2.15/2.01^h^	0.14	670^b^/11^b^/—	536/1/0.9	−5.29/−3.19	69
R-HDOBP	2.14/2.06^h^	0.08	662^b^/9^b^/—	534/2/1.2	−5.46/−3.08	69

a Lifetime of the delayed component.

b Measured in the neat film.

c Singlet energy (*E*_S_) measured in a diluted toluene solution at 298 K. Triplet energy (*E*_T_) determined in a diluted toluene solution at 77 K.

d Measured in doped film.

e Calculated from the onset wavelengths of the fluorescence (298 K) and phosphorescence (77 K) spectra of the emitter in the host.

f Estimated according to the 0–0 transition of fluorescence and phosphorescence spectra, respectively.

g Calculated by TD-DFT.

h Estimated from the fluorescence spectrum at 298 K and phosphorescence spectrum at 77 K (in the neat film).

**Table 2:** Summary of OLED structures and performances of TADF emitters discussed in this review

Emitter	Host	Device structures	*λ*_EL_ [nm]	EQE [%]	CE [cd A^−1^]	PE [lm W^−1^]	*L*_max_ (cd m^−2^)	Color index	Ref.
TPA-DCPP	Non-doped	ITO/NPB (80 nm)/TCTA (5 nm)/TPA-DCPP (20 nm)/TPBi (30 nm)/LiF (0.5 nm)/Al	710	2.1	—	—	591	(0.70, 0.29)	30
POZ-DBPHZ	10 wt% POZ-DBPHZ:mTDATA	ITO/*m*-MTDATA (40 nm)/10% POZ-DBPHZ in *m*-MTDATA (30 nm)/TPBi (50 nm)/LiF (1 nm)/Al (100 nm)	741	*ca.* 5	—	—	—	—	31
APDC-DTPA	Non-doped	Preliminary thermally evaporated OLED devices	777	2.19	—	—		—	32
DPA-Ph-DBPzDCN	15 wt% DPA-Ph-DBPzDCN:mCPPy2PO	ITO/NPB (40 nm)/TCTA (5 nm)/15 wt% DPA-Ph-DBPzDCN:mCPPy2PO (20 nm)/B3PymPm (10 nm)/Bepp2 (30 nm)/LiF (1 nm)/Al (100 nm)	698	7.68	1.27	—	603	(0.68, 0.30)	33
DPA-Ph-DBPzDCN	20 wt% DPA-Ph-DBPzDCN:mCPPy2PO	ITO/NPB (40 nm)/TCTA (5 nm)/20 wt% DPA-Ph-DBPzDCN:mCPPy2PO (20 nm)/B3PymPm (10 nm)/Bepp2 (30 nm)/LiF (1 nm)/Al (100 nm)	708	5.53	0.61	—	605	(0.69, 0.30)	33
DPA-Ph-DBPzDCN	30 wt% DPA-Ph-DBPzDCN:mCPPy2PO	ITO/NPB (40 nm)/TCTA (5 nm)/30 wt% DPA-Ph-DBPzDCN:mCPPy2PO (20 nm)/B3PymPm (10 nm)/Bepp2 (30 nm)/LiF (1 nm)/Al (100 nm)	720	3.94	0.34	—	383	(0.70, 0.30)	33
DPA-Ph-DBPzDCN	50 wt% DPA-Ph-DBPzDCN:mCPPy2PO	ITO/NPB (40 nm)/TCTA (5 nm)/50 wt% DPA-Ph-DBPzDCN:mCPPy2PO (20 nm)/B3PymPm (10 nm)/Bepp2 (30 nm)/LiF (1 nm)/Al (100 nm)	732	2.40	0.14	—	258	(0.70, 0.29)	33
TPA-PZCN	Non-doped	ITO/HAT-CN (10 nm)/TAPC (40 nm)/TCTA (10 nm)/CBP (10 nm)/TPA-PZCN (20 nm)/TmPyPB (55 nm)/Liq (2 nm)/Al (120 nm)	680	5.3	1.4	1.3	—	(0.69, 0.30)	34
HANTA-tCz	10 wt% HANTA-tCz:mCPCN	ITO/PEDOT:PSS (35 nm)/mCPCN:10 wt% HANTA-tCz:mCPCN (45–50 nm)/TmPyPB (60 nm)/Liq (1.5 nm)/Al (100 nm)	682	1.2	0.34	0.10	—	(0.68, 0.32)	35
HANTA-tPCz	5 wt% HANTA-tPCz:mCPCN	ITO/PEDOT:PSS (35 nm)/mCPCN:5 wt% HANTA-tPCz:mCPCN (45–50 nm)/TmPyPB (60 nm)/Liq (1.5 nm)/Al (100 nm)	692	4.8	1.54	0.54	—	(0.66, 0.32)	35
DCPPr-TPA	Non-doped	ITO/HATCN (5 nm)/TAPC (50 nm)/TCTA (5 nm)/DCPPr-TPA (20 nm)/TmPyPB (40 nm)/LiF (1 nm)/Al	734	1.4	0.11	0.11	501	(0.70, 0.29)	36
DCPPr-β-NDPA	Non-doped	ITO/HATCN (5 nm)/TAPC (50 nm)/TCTA (5 nm)/DCPPr-β-NDPA (20 nm)/TmPyPB (40 nm)/LiF (1 nm)/Al	748	1.4	0.07	0.06	203	(0.68, 0.28)	36
DCPPr-α-NDPA	Non-doped	ITO/HATCN (5 nm)/TAPC (50 nm)/TCTA (5 nm)/DCPPr-α-NDPA (20 nm)/TmPyPB (40 nm)/LiF (1 nm)/Al	716	1.9	0.25	0.22	638	(0.69, 0.30)	36
DCPPr-DBPPA	Non-doped	ITO/HATCN (5 nm)/TAPC (50 nm)/TCTA (5 nm)/DCPPr-DBPPA (20 nm)/TmPyPB (40 nm)/LiF (1 nm)/Al	748	1.0	0.041	0.038	123	(0.70, 0.28)	36
TPA-PZTCN	10 wt% TPA-PZTCN:mCBP	ITO/HATCN (10 nm)/TAPC (20 nm)/10 wt% TPA-PZTCN:mCBP (60 nm)/T2T (10 nm)/BPyTP2 (50 nm)/Liq (2 nm)/Al	734	13.4	—	—	—	—	37
*p*CNQ-TPA	Non-doped	ITO/MoO_3_ (6 nm)/mCP (70 nm)/*p*CNQ-TPA (30 nm)/TPBi (60 nm)/LiF (1 nm)/Al	700	4.62	0.80	0.78	—	(0.70, 0.30)	38
CNPP-TPA	50% CNPP-TPA:CBP	ITO/MoO_3_ (6 nm)/NPB (50 nm)/CBP:50 wt% CNPP-TPA (20 nm)/DPEPO (5 nm)/TPBi (35 nm)/LiF (1 nm)/Al	684	8.69	2.76	2.17	2356	(0.68, 0.31)	39
CNPP-TPA	60% CNPP-TPA:CBP	ITO/MoO_3_ (6 nm)/NPB (50 nm)/CBP:60 wt% CNPP-TPA (20 nm)/DPEPO (5 nm)/TPBi (35 nm)/LiF (1 nm)/Al	688	6.80	1.85	1.06	3318	(0.68, 0.32)	39
CNPP-TPA	Non-doped	ITO/MoO_3_ (6 nm)/NPB (50 nm)/CNPP-TPA (20 nm)/DPEPO (5 nm)/TPBi (35 nm)/LiF (1 nm)/Al	744	0.66	0.49	0.26	168.6	(0.68, 0.29)	39
CNPP-TPA	50% CNPP-TPA:CBP	ITO/PEDOT:PSS (20 nm)/CBP:50 wt% CNPP-TPA (100 nm)/DPEPO (5 nm)/TPBi (40 nm)/LiF|Al	700	2.02	0.33	0.17	96.7	(0.69, 0.30)	39
AQTC-DTPA	10 wt% AQTC-DTPA:CBP	ITO/HAT-CN (10 nm)/TAPC (40 nm)/TCTA (10 nm)/CBP:10 wt% AQTC-DTPA (20 nm)/PO-T2T (60 nm)/Liq (2 nm)/Al (120 nm)	694	9.28	—	—	—	—	40
AQTC-DTPA	20 wt% AQTC-DTPA:CBP	ITO/HAT-CN (10 nm)/TAPC (40 nm)/TCTA (10 nm)/CBP:20 wt% AQTC-DTPA (20 nm)/PO-T2T (60 nm)/Liq (2 nm)/Al (120 nm)	740	3.88	—	—	—	—	40
AQTC-DTPA	30 wt% AQTC-DTPA:CBP	ITO/HAT-CN (10 nm)/TAPC (40 nm)/TCTA (10 nm)/CBP:30 wt% AQTC-DTPA (20 nm)/PO-T2T (60 nm)/Liq (2 nm)/Al (120 nm)	754	2.50	—	—	—	—	40
AQTC-DTPA	40 wt% AQTC-DTPA:CBP	ITO/HAT-CN (10 nm)/TAPC (40 nm)/TCTA (10 nm)/CBP:40 wt% AQTC-DTPA (20 nm)/PO-T2T (60 nm)/Liq (2 nm)/Al (120 nm)	770	1.51	—	—	—	—	40
AQTC-DTPA	50 wt% AQTC-DTPA:CBP	ITO/HAT-CN (10 nm)/TAPC (40 nm)/TCTA (10 nm)/CBP:50 wt% AQTC-DTPA (20 nm)/PO-T2T (60 nm)/Liq (2 nm)/Al (120 nm)	788	0.76	—	—	—	—	40
AQTC-DTPA	60 wt% AQTC-DTPA:CBP	ITO/HAT-CN (10 nm)/TAPC (40 nm)/TCTA (10 nm)/CBP:60 wt% AQTC-DTPA (20 nm)/PO-T2T (60 nm)/Liq (2 nm)/Al (120 nm)	810	0.51	—	—	—	—	40
AQTC-DTPA	70 wt% AQTC-DTPA:CBP	ITO/HAT-CN (10 nm)/TAPC (40 nm)/TCTA (10 nm)/CBP:70 wt% AQTC-DTPA (20 nm)/PO-T2T (60 nm)/Liq (2 nm)/Al (120 nm)	828	0.41	—	—	—	—	40
AQTC-DTPA	80 wt% AQTC-DTPA:CBP	ITO/HAT-CN (10 nm)/TAPC (40 nm)/TCTA (10 nm)/CBP:80 wt% AQTC-DTPA (20 nm)/PO-T2T (60 nm)/Liq (2 nm)/Al (120 nm)	852	0.30	—	—	—	—	40
AQTC-DTPA	Non-doped	ITO/HAT-CN (10 nm)/TAPC (40 nm)/TCTA (10 nm)/AQTC-DTPA (30 nm)/PO-T2T (60 nm)/Liq (2 nm)/Al (120 nm)	894	0.23	—	—	—	—	40
AQTC-DTPA	30 nm AQTC-DTPA	ITO/HAT-CN (10 nm)/TAPC (40 nm)/TCTA (10 nm)/AQTC-DTPA (30 nm)/TmPyPB (60 nm)/Liq (2 nm)/Al (120 nm)	908	0.17	—	—	—	—	40
AQTC-DTPA	40 nm AQTC-DTPA	ITO/HAT-CN (10 nm)/TAPC (40 nm)/TCTA (10 nm)/AQTC-DTPA (30 nm)/TmPyPB (60 nm)/Liq (2 nm)/Al (120 nm)	910	0.22	—	—	—	—	40
TPA-QCN	30 wt% TPA-QCN:TPBi	ITO/NPB (65 nm)/mCP (5 nm)/30 wt% TPA-QCN:TPBi (20 nm)/B3PyMPM (30 nm)/LiF (1 nm)/Al (100 nm)	700	9.4	1.6	1.6	1371	(0.68, 0.31)	41
TPA-QCN	Non-doped	ITO/NPB (65 nm)/mCP (5 nm)/TPA-QCN (20 nm)/B3PyMPM (30 nm)/LiF (1 nm)/Al (100 nm)	728	3.9	0.3	0.3	205	(0.69, 0.31)	41
TPA-PPDCN	20 wt% TPA-PPDCN:CBP	ITO/TAPC (40 nm)/mCP (5 nm)/20 wt% TPA-PPDCN:CBP (20 nm)/B3PyMPM (50 nm)/LiF (1 nm)/Al	692	16.4	3.1	2.7	923	(0.70, 0.30)	42
TPAAP	15 wt% TPAAP:TPBi	ITO/HATCN (5 nm)/NPB (70 nm)/TCTA (5 nm)/TPBi:15 wt% TPAAP (30 nm)/TPBi (40 nm)/LiF (1 nm)/Al (150 nm)	700	14.1	—	—	—	—	43
TPAAP	Non-doped	ITO/HATCN (5 nm)/NPB (70 nm)/TCTA (5 nm)/TPAAP (30 nm)/TPBi (40 nm)/LiF (1 nm)/Al (150 nm)	765	5.1	—	—	—	—	43
CAT-1	Non-doped	Preliminary thermally evaporated OLED devices	904	*ca.* 0.019	—	—	—	—	44
TPAAQ	Non-doped	ITO/HATCN (5 nm)/NPB (70 nm)/TCTA (5 nm)/TPAAQ (30 nm)/TPBi (40 nm)/LiF (1 nm)/Al (150 nm)	711	3.5	—	—	—	—	43
mDPBPZ-PXZ	Non-doped	ITO/TAPC (35 nm)/TCTA (10 nm)/mCP (10 nm)/emitters (20 nm)/TmPyPB (45 nm)/LiF (1 nm)/Al	680	5.2	2.8	2.3	—	(0.68, 0.32)	45
DCPA-TPA	10 wt% DCPA-TPA:CBP	ITO/MoO_3_ (2.5 nm)/NPB (35 nm)/TCTA (10 nm)/DCPA-TPA (30 or 40 nm)/3TPYMB (10 nm)/Bpy-TP2(50 nm)/Liq (2 nm)/Al (120 nm)	704	2.7	—	—	—	—	46
DCPA-TPA	20 wt% DCPA-TPA:CBP	ITO/MoO_3_ (2.5 nm)/NPB (35 nm)/TCTA (10 nm)/DCPA-TPA (30 or 40 nm)/3TPYMB (10 nm)/Bpy-TP2(50 nm)/Liq (2 nm)/Al (120 nm)	734	1.72	—	—	—	—	46
DCPA-TPA	30 wt% DCPA-TPA:CBP	ITO/MoO_3_ (2.5 nm)/NPB (35 nm)/TCTA (10 nm)/DCPA-TPA (30 or 40 nm)/3TPYMB (10 nm)/Bpy-T-P2(50 nm)/Liq (2 nm)/Al (120 nm)	752	1.40	—	—	—	—	46
DCPA-TPA	Non-doped	ITO/MoO_3_ (2.5 nm)/NPB (35 nm)/TCTA (10 nm)/DCPA-TPA (30 or 40 nm)/3TPYMB (10 nm)/Bpy-TP2(50 nm)/Liq (2 nm)/Al (120 nm)	838	0.58	—	—	—	—	46
DCPA-BBPA	10 wt% DCPA-BBPA:CBP	ITO/MoO_3_ (2.5 nm)/NPB (35 nm)/TCTA (10 nm)/DCPA-BBPA (30 or 40 nm)/3TPYMB (10 nm)/Bpy-TP2 (50 nm)/Liq (2 nm)/Al (120 nm)	744	2.06	—	—	—	—	46
DCPA-BBPA	20 wt% DCPA-BBPA:CBP	ITO/MoO_3_ (2.5 nm)/NPB (35 nm)/TCTA (10 nm)/DCPA-BBPA (30 or 40 nm)/3TPYMB (10 nm)/Bpy-TP2 (50 nm)/Liq (2 15 nm)/Al (120 nm)	774	1.30	—	—	—	—	46
DCPA-BBPA	30 wt% DCPA-BBPA:CBP	ITO/MoO_3_ (2.5 nm)/NPB (35 nm)/TCTA (10 nm)/DCPA-BBPA (30 or 40 nm)/3TPYMB (10 nm)/Bpy-TP2 (50 nm)/Liq (2 nm)/Al (120 nm)	812	0.50	—	—	—	—	46
DCPA-BBPA	Non-doped	ITO/MoO_3_ (2.5 nm)/NPB (35 nm)/TCTA (10 nm)/DCPA-BBPA (30 or 40 nm)/3TPYMB (10 nm)/Bpy-TP2 (50 nm)/Liq (2 nm)/Al (120 nm)	916	0.07	—	—	—	—	46
TPAAZ	1 wt% TPAAZ:CBP	ITO/HATCN (5 nm)/TAPC (60 nm)/TCTA (5 nm)/1 wt% TPAAZ:CBP (30 nm)/B3PYMPM (60 nm)/LiF (1 nm)/Al (150 nm)	722	1.35	—	—	—	—	47
TPAAZ	Non-doped	ITO/HATCN (5 nm)/TAPC (60 nm)/TCTA (5 nm)/TPAAZ (30 nm)/B3PYMPM (60 nm)/LiF (1 nm)/Al (150 nm)	1010	0.003	—	—	—	—	47
dpTPAAP	5 wt% dpTPAAP:TPBi	ITO/HATCN (5 nm)/NPB (70 nm)/TCTA (10 nm)/TPBi:5 wt% dpTPAAP (30 nm)/TPBi (60 nm)/LiF (1 nm)/Al (150 nm)	710	17	—	—	—	—	48
dpTPAAP	15 wt% dpTPAAP:TPBi	ITO/HATCN (5 nm)/NPB (70 nm)/TCTA (10 nm)/TPBi:15 wt% dpTPAAP (30 nm)/TPBi (60 nm)/LiF (1 nm)/Al (150 nm)	730	13	—	—	—	—	48
dpTPAAP	30 wt% dpTPAAP:TPBi	ITO/HATCN (5 nm)/NPB (70 nm)/TCTA (10 nm)/TPBi:30 wt% dpTPAAP (30 nm)/TPBi (60 nm)/LiF (1 nm)/Al (150 nm)	752	6.8	—	—	—	—	48
dpTPAAZ	5 wt% dpTPAAZ:TPBi	ITO/HATCN (5 nm)/NPB (70 nm)/TCTA (10 nm)/TPBi:5 wt% dpTPAAZ (30 nm)/TPBi (60 nm)/LiF (1 nm)/Al (150 nm)	757	1.2	—	—	—	—	48
SDPA-APDC	30 wt% SDPA-ADPC:CBP	ITO/MoO_3_ (2.5 nm)/TAPC (30 nm)/TCTA (10 nm)/CBP:30 wt% emitter (20 nm)/B3PyMPM (60 nm)/Liq (2 nm)/Al (120 nm)	696	10.75	—	—	—	(0.69, 0.30)	49
SDPA-APDC	50 wt% SDPA-ADPC:CBP	ITO/MoO_3_ (2.5 nm)/TAPC (30 nm)/TCTA (10 nm)/CBP:50 wt% emitter (20 nm)/B3PyMPM (60 nm)/Liq (2 nm)/Al (120 nm)	728	5.05	—	—	—	—	49
SDPA-APDC	Non-doped	ITO/MoO_3_ (2.5 nm)/TAPC (30 nm)/TCTA (10 nm)/SDPA-ADPC (20 nm)/B3PyMPM (60 nm)/Liq (2 nm)/Al (120 nm)	782	2.55	—	—	—	—	49
CN-TPA	7.5 wt% CN-TPA:CBP	ITO/HAT-CN (10 nm)/TAPC (50 nm)/CBP:7.5 wt% CN-TPA (20 nm)/B3PYMPM (60 nm)/Liq (2 nm)/Al (120 nm)	688	18.41	5.02	5.84	235	(0.68, 0.32)	51
CN-TPA	10 wt% CN-TPA:CBP	ITO/HAT-CN (10 nm)/TAPC (50 nm)/CBP:10 wt% CN-TPA (20 nm)/B3PYMPM (60 nm)/Liq (2 nm)/Al (120 nm)	698	15.05	4.77	5.54	204	(0.69, 0.31)	51
TPAAP-D	Non-doped	ITO/HAT-CN (10 nm)/TAPC (40 nm)/TCTA (10 nm)/CBP:TPAAP-D (20 nm)/TmPyPB (55 nm)/Liq (2 nm)/Al (120 nm)	766	2.5	0.07	0.08	—	(0.69, 0.29)	52
TPAAQ-D	Non-doped	ITO/HAT-CN (10 nm)/TAPC (40 nm)/TCTA (10 nm)/CBP:TPAAQ-D (20 nm)/TmPyPB (55 nm)/Liq (2 nm)/Al (120 nm)	717	3.0	0.18	0.19	—	(0.70, 0.30)	52
CAT-TPE	15 wt% CAT-TPE:TPBi	ITO/PEDOT:PSS (40 nm)/PVK (10 nm)/15 wt% CAT-TPE in TPBi (40 nm)/10 wt% TPBi:TSPO1 (10 nm)/TSPO1 (40 nm)/LiF (0.8 nm)/Al (100 nm)	720	0.8	—	—	—	—	53
CAT-TPE	Pristine	ITO/PEDOT:PSS (40 nm)/PVK (10 nm)/CAT-TPE in TPBi (40 nm)/TPBi:TSPO1 (10 nm)/TSPO1 (40 nm)/LiF (0.8 nm)/Al (100 nm)	755	0.2	—	—	—	—	53
TPA–CN–N4	12 wt% TPA–CN–N4:mCPCN	ITO (85 nm)/MoO_3_ (1 nm)/TAPC (115 nm)/mCP (10 nm)/mCPCN:12 wt% TPA–CN–N4 (20 nm)/3TPYMB (75 nm)/LiF (1 nm)/Al	689	21.0	4.8	5.3	285.4	—	54
TPA–CN–N4-2PY	9 wt% TPA–CN–N4-2PY:mCPCN	ITO (85 nm)/MoO_3_ (1 nm)/TAPC (120 nm)/mCP (10 nm)/mCPCN:9 wt% TPA–CN–N4-2PY:mCPCN (20 nm)/3TPYMB (75 nm)/LiF (1 nm)/Al	712	21.9	4.2	4.1	88.5	(0.67, 0.32)	54
MPPA:MCBP (1 : 4)	Non-doped	ITO/PEDOT:PSS/MPPA:MCBP (1 : 4)/TPBi/Cs_2_CO_3_/Al	710	0.11	—	—	—	(0.69, 0.31)	55
TPA-cNDI	10 wt% TPA-cNDI:CBP	ITO/NPB (40 nm)/TAPC (10 nm)/10% TPA-cNDI co CBP (20 nm)/TPBi (10 nm)/PO-T2T (40 nm)/LiF (1 nm)/Al (100 nm)	*ca.* 735	2.4	—	—	—	—	56
BDC-1	2 wt% BDC-2:CBP	ITO (100 nm)/PEDOT:PSS (45 nm)/2 wt% BDC-2:CBP (∼80 nm)/DPEPO (10 nm)/TPBi (55 nm)/LiF (1 nm)/Al (100 nm)	705	8.41	—	—	—	—	57
BDC-1	4 wt% BDC-2:CBP	ITO (100 nm)/PEDOT:PSS (45 nm)/4 wt% BDC-2:CBP (∼80 nm)/DPEPO (10 nm)/TPBi (55 nm)/LiF (1 nm)/Al (100 nm)	716	8.53	—	—	—	—	57
BDC-1	6 wt% BDC-2:CBP	ITO (100 nm)/PEDOT:PSS (45 nm)/6 wt% BDC-2:CBP (∼80 nm)/DPEPO (10 nm)/TPBi (55 nm)/LiF (1 nm)/Al (100 nm)	721	9.69	—	—	—	—	57
BDC-1	7 wt% BDC-2:CBP	ITO (100 nm)/PEDOT:PSS (45 nm)/7 wt% BDC-2:CBP (∼80 nm)/DPEPO (10 nm)/TPBi (55 nm)/LiF (1 nm)/Al (100 nm)	730	8.09	—	—	—	—	57
BDC-1	8 wt% BDC-2:CBP	ITO (100 nm)/PEDOT:PSS (45 nm)/8 wt% BDC-2:CBP (∼80 nm)/DPEPO (10 nm)/TPBi (55 nm)/LiF (1 nm)/Al (100 nm)	732	5.56	—	—	—	—	57
BDC-1	10 wt% BDC-2:CBP	ITO (100 nm)/PEDOT:PSS (45 nm)/10 wt% BDC-2:CBP (∼80 nm)/DPEPO (10 nm)/TPBi (55 nm)/LiF (1 nm)/Al (100 nm)	734	3.19	—	—	—	—	57
BDC-1	15 wt% BDC-2:CBP	ITO (100 nm)/PEDOT:PSS (45 nm)/15 wt% BDC-2:CBP (∼80 nm)/DPEPO (10 nm)/TPBi (55 nm)/LiF (1 nm)/Al (100 nm)	743	2.03	—	—	—	—	57
BDC-1	20 wt% BDC-2:CBP	ITO (100 nm)/PEDOT:PSS (45 nm)/20 wt% BDC-2:CBP (∼80 nm)/DPEPO (10 nm)/TPBi (55 nm)/LiF (1 nm)/Al (100 nm)	750	1.44	—	—	—	—	57
BDC-1	40 wt% BDC-2:CBP	ITO (100 nm)/PEDOT:PSS (45 nm)/40 wt% BDC-2:CBP (∼80 nm)/DPEPO (10 nm)/TPBi (55 nm)/LiF (1 nm)/Al (100 nm)	761	0.71	—	—	—	—	57
BDC-1	60 wt% BDC-2:CBP	ITO (100 nm)/PEDOT:PSS (45 nm)/60 wt% BDC-2:CBP (∼80 nm)/DPEPO (10 nm)/TPBi (55 nm)/LiF (1 nm)/Al (100 nm)	771	0.34	—	—	—	—	57
BDC-1	Non-doped	ITO (100 nm)/PEDOT:PSS (45 nm)/BDC-2 (∼80 nm)/DPEPO (10 nm)/TPBi (55 nm)/LiF (1 nm)/Al (100 nm)	782	0.27	—	—	—	—	57
BDC-2	40 wt% BDC-3:CBP	ITO (100 nm)/PEDOT:PSS (45 nm)/40 wt% BDC-3:CBP (∼80 nm)/DPEPO (10 nm)/TPBi (55 nm)/LiF (1 nm)/Al (100 nm)	796	0.30	—	—	—	—	58
TPAM-BF_2_	6 wt% CBP:TPAM-BF_2_	ITO/PEDOT:PSS (43 nm)/CBP:6 wt% TPAM-BF_2_ (80 nm)/B3PYMPM (80 nm)/LiF (1 nm)/Al (140 nm)	737	6.5 ± 0.3%	—	—	—	—	60
TPAM-BF_2_	10 wt% CBP:TPAM-BF_2_	ITO/PEDOT:PSS (43 nm)/CBP:10 wt% TPAM-BF_2_ (80 nm)/B3PYMPM (80 nm)/LiF (1 nm)/Al (140 nm)	750	5.9 ± 0.1%	—	—	—	—	60
TPAM-BF_2_	15 wt% CBP:TPAM-BF_2_	ITO/PEDOT:PSS (43 nm)/CBP:15 wt% TPAM-BF_2_ (80 nm)/B3PYMPM (80 nm)/LiF (1 nm)/Al (140 nm)	758	4 ± 0.1%	—	—	—	—	60
TPAM-BF_2_	20 wt% CBP:TPAM-BF_2_	ITO/PEDOT:PSS (43 nm)/CBP:20 wt% TPAM-BF_2_ (80 nm)/B3PYMPM (80 nm)/LiF (1 nm)/Al (140 nm)	759	3.9 ± 0.1%	—	—	—	—	60
TPAM-BF_2_	30 wt% CBP:TPAM-BF_2_	ITO/PEDOT:PSS (43 nm)/CBP:30 wt% TPAM-BF_2_ (80 nm)/B3PYMPM (80 nm)/LiF (1 nm)/Al (140 nm)	765	2.3 ± 0.1%	—	—	—	—	60
CzTCF	Non-doped	ITO/PEDOT:PSS (70 nm)/PVK (10 nm)/CzTCF (30 nm)/DPEPO (10 nm)/TmPyPB (50 nm)/Liq (1 nm)/Al (100 nm)	683	0.3	*ca.* 0.05	*ca.* 0.02	*ca.* 50	—	61
tBCzTCF	Non-doped	ITO/PEDOT:PSS (70 nm)/PVK (10 nm)/tBCzTCF (30 nm)/DPEPO (10 nm)/TmPyPB (50 nm)/Liq (1 nm)/Al (100 nm)	715	*ca.* 0.2	*ca.* 0.02	*ca.* 0.01	*ca.* 50	—	61
PIBz-3-PTZ	Non-doped	ITO/HATCN (5 nm)/TAPC (25 nm)/TCTA (15 nm)/PIBz-3-PTZ (20 nm)/TPBi (40 nm)/LiF (1 nm)/Al (100 nm)	672	2.02	0.70	0.48	3403	(0.67, 0.32)	63
TPACNBz	30 wt% TPACNBz:CBP	ITO/PEDOT:PSS (40 nm)/NPB (30 nm)/30 wt% EML:CBP (45 nm)/TmPyPB (35 nm)/LiF (0.5 nm)/Al (100 nm)	712	6.57	—	—	—	(0.68, 0.29)	64
R-TBN	CBP:30 wt% Ir(mphmq)_2_tmd:1 wt% R-TBN	ITO/HATCN (10 nm)/TAPC (70 nm)/TCTA (10 nm)/EMLs (25 nm)/CzPhPy (10 nm)/B4PyMPM (45 nm)/LiF (0.5 nm)/Al (150 nm)	684	23.9	—	—	—	(0.72, 0.28)	68
R-TBN	CBP:30 wt% Ir(mphmq)_2_tmd:2 wt% R-TBN	ITO/HATCN (10 nm)/TAPC (70 nm)/TCTA (10 nm)/EMLs (25 nm)/CzPhPy (10 nm)/B4PyMPM (45 nm)/LiF (0.5 nm)/Al (150 nm)	684	26.1	—	—	—	(0.72, 0.28)	68
R-TBN	CBP:30 wt% Ir(mphmq)_2_tmd:3 wt% R-TBN	ITO/HATCN (10 nm)/TAPC (70 nm)/TCTA (10 nm)/EMLs (25 nm)/CzPhPy (10 nm)/B4PyMPM (45 nm)/LiF (0.5 nm)/Al (150 nm)	686	27.6	—	—	—	(0.72, 0.28)	68
R-TBN	CBP:30 wt% Ir(mphmq)_2_tmd:4 wt% R-TBN	ITO/HATCN (10 nm)/TAPC (70 nm)/TCTA (10 nm)/EMLs (25 nm)/CzPhPy (10 nm)/B4PyMPM (45 nm)/LiF (0.5 nm)/Al (150 nm)	686	21.7	—	—	—	(0.72, 0.28)	68
R-TBN	CBP:2 wt% R-TBN	ITO/HATCN (10 nm)/TAPC (60 nm)/TCTA (10 nm)/EML (30 nm)/CzPhPy (10 nm)/B4PyMPM (50 nm)/LiF (0.5 nm)/Al (150 nm)	686	2.7	—	—	—	(0.72, 0.28)	68
R-DOBP	Non-doped	ITO/PEDOT:PSS (70 nm)/R-DOBP (50 nm)/DPEPO (10 nm)/TmPyPB (50 nm)/Liq (1 nm)/Al (100 nm)	716	1.9	—	—	—	—	69
R-HDOBP	Non-doped	ITO/PEDOT:PSS (70 nm)/R-HDOBP (50 nm)/DPEPO (10 nm)/TmPyPB (50 nm)/Liq (1 nm)/Al (100 nm)	700	0.7	—	—	—	—	69

## Approaches to constructing NIR TADF emitters

According to spin quantum theory, the ratio of singlet and triplet excitons is 1 : 3 during the electrical excitation processes. As a result, traditional fluorescent materials can only use the radiation energy from the S_1_ state with 25% internal quantum efficiency. This also means that the remaining 75% of energy from triplet excitons is lost. Therefore, it is important to acquire more triplet energy to improve the OLED efficiencies. For this purpose, heavy-metal atoms are usually introduced to enhance spin–orbit coupling for promoting the RISC process to obtain higher exciton utilization. In this way, both triplet and singlet excitons can be harvested, which makes it possible for OLEDs to theoretically achieve 100% IQE. Nevertheless, heavy metals are rare and expensive. Researchers tried to develop new strategies to utilize triplet excitons, such as triplet–triplet annihilation, hybridized local and charge-transfer states, and TADF.

In order to achieve TADF, researchers often design molecules with twisted structures to reduce the overlapping between the highest occupied molecular orbital (HOMO) and the lowest unoccupied molecular orbital (LUMO). In addition, bulky and twisted structures could restrict the conjugation which could result in an efficient intramolecular charge transfer (ICT) process.^24^ In general, narrowing the bandgap, *i.e.*, reducing the gap between the LUMO and HOMO energy levels, is one of the basic molecular design principles for donor–acceptor (D–A) type organic TADF materials. It has been proved to be an effective way to tune molecular energy levels because bandgaps could be easily adjusted by changing the electron density on the donor and acceptor units.^25^ Considering that an appropriate D and A could minimize the singlet–triplet energy gap Δ*E*_ST_, it is essential to achieve efficient reverse ISC (RISC) processes. Meanwhile, the highly twisted structure required for small Δ*E*_ST_ is not beneficial for red-shifting the emission. Thus, it is important to first explore acceptor moieties with strong electron-withdrawing ability. According to our review, rigid and planar fused heterocycles have been widely used for acceptors. Both Δ*E*_ST_ and fluorescence efficiencies should be taken into consideration. However, luminescence efficiency is often low due to the small coupling between S_1_ and S_0_ from the separated HOMO and LUMO. But once the high probability of coupling of vibrational manifolds between the ground states and the excited states with the red-shifted emission wavelengths exists, the non-radiative transition rates would be increased rapidly.^26^ Therefore, the inherent contradiction between small Δ*E*_ST_ and high fluorescence efficiency results in a dilemma in designing NIR TADF molecules.

In addition to the general design rules of TADF emitters, employing rigid donor or acceptor structures is preferred for higher EQEs by suppressing non-radiative transition. Rigid groups can efficiently avoid molecular motions by freezing the molecular structures. Additionally, the rigid moieties could narrow the emission spectra, which also increases the EQE of the NIR TADF emitters. In the reported NIR TADF emitters, only a few D moieties are employed, such as triphenylamine, carbazole, phenoxazine and their derivatives, while there are a large variety of A groups that can be selected to manipulate the molecular properties, which has also become a hotspot nowadays. In general, acceptor groups for NIR TADF molecules have focused on pyrazine derivatives, boron–nitrogen compounds, difluoroboron compounds and so on. The development of new acceptor materials is beneficial for improving device performance and broadening the application of NIR TADF materials. Thus, the development of new functionalized acceptor units with simple structures is still imperative.

Mostly, NIR OLEDs based on TADF emitters usually employ doping strategies to minimize possible annihilation problems of the emitters during the light-emitting process. However, doped devices are prone to phase separation and crystallization during the fabrication process, which affects the stability of the device and leads to a frustrating low energy utilization rate. Moreover, the multi-layer structure increases the difficulty in the fabrication process.^27,28^ Thus, researchers are trying to develop high-efficiency light-emitting materials for non-doped OLED luminescent layers. For this purpose, light-emitting molecules must be well designed, which means that they should have a subtle configuration as well as the optimal optoelectronic properties. Firstly, the packing form of the molecules in the aggregation state can effectively inhibit emission quenching to achieve high-efficiency carrier transport. Secondly, the radiative transition rate should have an overwhelming advantage over non-radiation to achieve high photoluminescence quantum yield (PLQY). Moreover, rapid charge recombination and exciton radiation are beneficial for avoiding exciton accumulation.^29^ Therefore, we also introduced some non-doped OLED luminescent materials in this review.

## NIR TADF emitters and their OLEDs

**Scheme 1:**
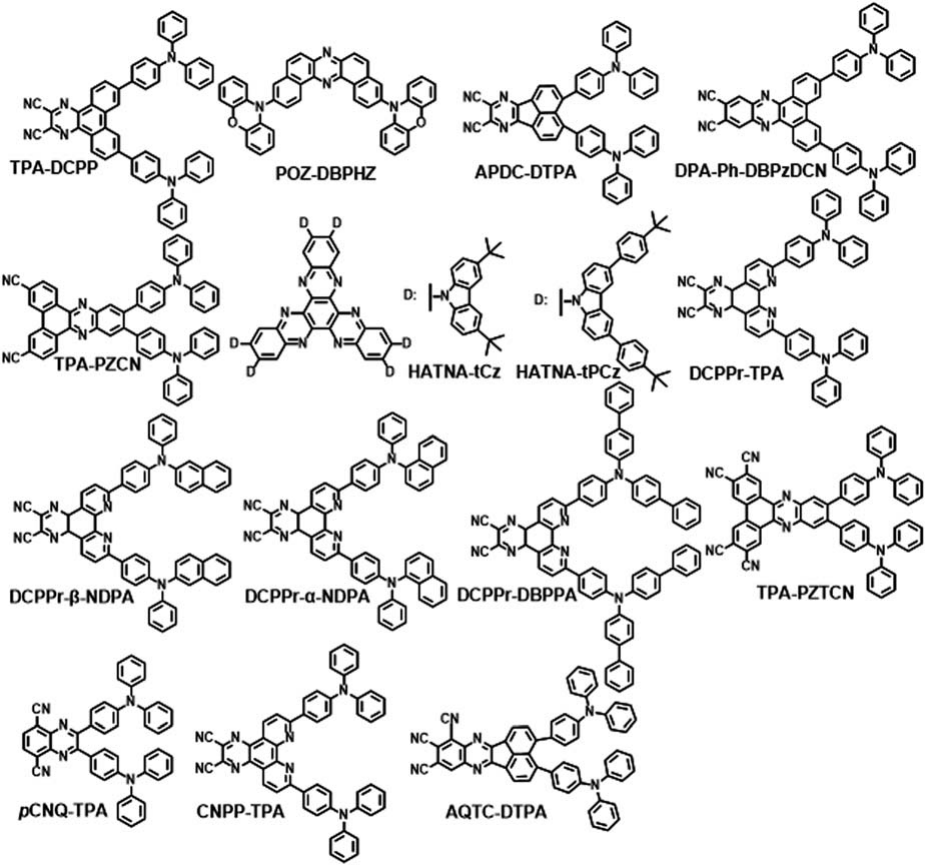
TADF molecules based on pyrazine derivatives (with multiple donors).

**Scheme 2:**
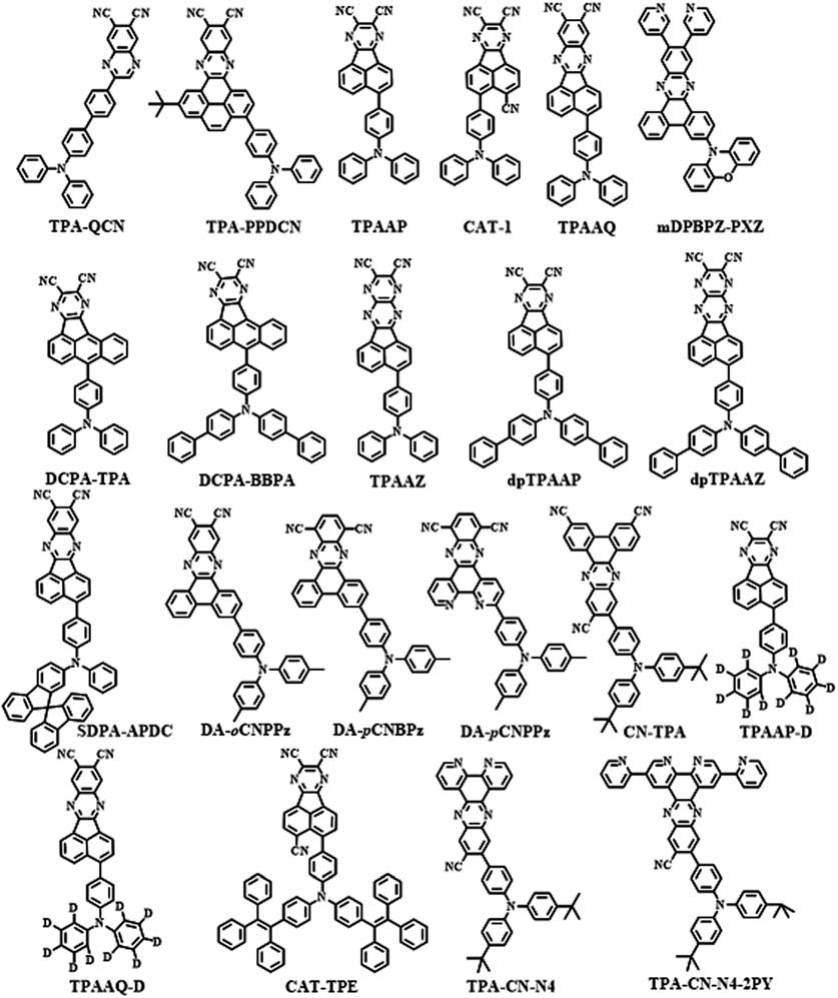
TADF molecules based on pyrazine derivatives (with a single donor).

As mentioned above, it is important to get a small singlet energy level to realize NIR emission in D–A type TADF emitters. The degree of charge transfer (CT) character is decided by donor and acceptor moieties. Therefore, either strong electron-donating or electron-withdrawing groups are required to realize NIR emission. It was found that pyrazine derivatives can act as excellent acceptors for NIR TADF molecules according to Schemes 1 and 2. Based on the analysis of existing studies, it can be concluded that many of the electron donors of NIR TADF molecules based on pyrazine are triphenylamine and its derivatives. To facilitate the comparison of their photophysical properties, we divided them into derivatives with multiple donors (Scheme 1) and those with a single donor (Scheme 2).

## Pyrazine derivatives with multiple donors

The first NIR TADF molecule, TPA-DCPP (Scheme 1), was reported by Wang *et al.* in 2015. A pyrazine derivative with an extended conjugation structure, 2,3-dicyanopyrazino phenanthrene (DCPP), was chosen as the electron-withdrawing moiety. An effective HOMO–LUMO separation and partial orbital overlaps deriving from the D–π–A–π–D configuration ensure Δ*E*_ST_ as small as 0.13 eV and a large *k*_f_ value of 9.0 × 10^−7^ s^−1^ for TPA-DCPP. It showed a broad NIR emission with the maximum at 708 nm and a remarkable *Φ*_PL_ value of 14% in the neat film. The non-doped OLED device based on TPA-DCPP exhibited a maximum EQE of 2.1% and an EL peak at 710 nm with CIE coordinates of (0.70, 0.29) (as shown in Fig. 1(a) and (b)).^30^

In addition to typical triphenylamine and diphenylamine, pyrazine-acceptor based NIR TADF materials could also employ carbazole (Cz) derivatives and phenoxazine (POZ) as donors. A new acceptor unit core, dibenzo-[*a*,*j*]phenazine (DBPHZ), was designed in 2016 and one of its derivatives, POZ-DBPHZ (Scheme 1), was constructed by an oxidative skeletal rearrangement of 1,1′-binaphthalene-2,2′-diamines. POZ-DBPOHZ showed a small energy gap of 0.02 eV as the effective HOMO/LUMO separation. It was found that the exciplex formed with *m*-MTDATA (host material) and POZ-DBPHZ showed NIR emission with the emission wavelength at efficient TADF by triplet state coupling of acceptors.^31^

To improve both the efficiency and color purity of NIR TADF OLEDs, an acenaphtho[1,2-*b*]pyrazine-8,9-dicarbonitrile acceptor (APDC) core was combined with two diphenylamine donor units to design the wedge-shaped D–π–A–π–D emitter APDC-DTPA (Scheme 1) in 2017. The new acceptor APDC exhibited strong electron-withdrawing ability due to the formation of the aromatic cyclopentadienide structure with six π-electrons in the central fluoranthene core. The non-doped device based on APDC-DTPA exhibited NIR emission with a maximum of 777 nm and a high EQE of 2.19%. In addition, 10 wt% and 20 wt% doped devices showed excellent EQEs of 10.19% (emission peak at 693 nm) and 9.70% (emission peak at 696 nm), respectively.^32^

Based on DCPP, Wang *et al.* developed DBPzDCN by extending the π-conjugated length and increasing the electron-withdrawing ability. Due to the relatively small twist angle, the orbital distribution of DPA-Ph-DBPzDCN (Scheme 1) has a partial orbital overlap of the HOMO/LUMO on the DBPzDCN acceptor, leading to a high oscillator strength of 0.0744, which would boost the radiative fluorescence rate.

The reducing LUMO indicated that extending the π-conjugation led to an increase in electron-withdrawing ability. Thus, DPA-Ph-DBPzDCN was regulated to a redshift emission with the maximum of 765 nm in the non-doped thin film. Notably, the doped OLED (Fig. 4(a)) device achieved an emission with the maximum at 698 nm and CIE coordinates of (0.68, 0.30), as well as a decent EQE of 7.68%.^33^

Liao and coworkers developed a novel TADF emitter in which a new acceptor, dibenzo[*a*,*c*]phenazine-3,6-dicarbonitrile (PZCN), was used. The large and planar backbone of the emitter was designed for suppressing the nonradiative transition. TPA-PZCN (as shown in Scheme 2) showed a high *Φ*_PL_ of 97% and an effective RISC process. The corresponding non-doped device exhibited an NIR emission peaking at 680 nm and a high EQE of 5.3%.^34^

Yang *et al.* reported two typical TADF emitters by utilizing multiple Cz derivatives as electron donors in 2019, named HATNA-tCz and HATNA-tPCz, respectively (Scheme 1). 5,6,11,12,17,18-Hexaazatrinaphthylene (HATNA) was regarded as a very important core for TADF receptors among pyrazine derivatives too. It was constructed with a large and rigid π-conjugated structure ensuring strong electron-withdrawing ability. The introduction of a peripheral *tert*-butyl unit provided great solubility with promising applications in solution-processed OLEDs. Doped devices based on HATNA-tCz and HATNA-tPCz (Fig. 4(b)) displayed EL emissions with the maxima of 682 and 692 nm, respectively.^35^ This work also provided an easy method with low cost to construct efficient solution-processable NIR TADF emitters.

To further enhance molecular rigidity and planarity, four new TADF molecules with a strong electron-withdrawing pyrazino[2,3-*f*][1,10]phenanthroline-2,3-dicarbonitrile (DCPPr) core and electron-donating triarylamine (Ar_3_N) moieties, including *N*,*N*-diphenylnaphthalen-1-amine (α-NDPA), *N*,*N*-diphenylnaphthalen-2-amine (β-NDPA), triphenylamine (TPA) and *N*,*N*-di([1,1′-biphenyl]-4-yl)phenylamine (DBPPA), were reported. The nitrogen atoms of DCPPr in pyridine rings were able to form hydrogen bonds with the adjacent phenyl rings of Ar_3_N, which largely improved the emission efficiency and horizontal orientation. All of the four new TADF molecules showed NIR emissions (692–710 nm) in neat films. In addition, high-performance non-doped OLEDs with NIR light were attained based on these molecules.^36^

Researchers also paid attention to the roll-off problems of EQE in NIR-OLEDs besides high EQE. More C≡N groups were used to construct a new receptor named PZTCN, based on which TPA-PZTCN (Scheme 1), with a well suppressed roll-off behavior of EQE (EQE > 10% at 1 mA cm^−2^), exhibited intense NIR EL (EQE_max_: 13.4% ± 0.8%) at a peak wavelength of 734 nm by harvesting triplet excitons as delayed fluorescence. It was outstanding compared with the previously reported NIR-TADF-OLEDs emitting beyond 700 nm (EQE < 6% at 1 mA cm^−2^), which could be attributed to the small RISC rate constant. TPA-PZTCN can achieve a deeper NIR fluorophore to achieve a peak wavelength of approximately 900 nm, resulting in an EQE of over 1% in a TADF-sensitized NIR OLED with high operational device durability.^37^

**Fig. 2:**
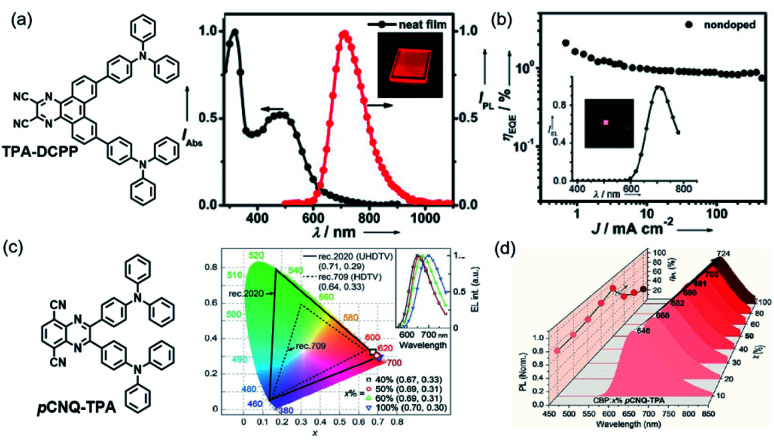
(a) The absorption (black) and PL (red) spectra of TPA-DCPP in a neat film. Inset: image of the neat film under UV irradiation (*λ*_ex_ = 365 nm). Copyright 2015, John Wiley & Sons. (b) EQE *versus* current density characteristics of the TPA-DCPP based non-doped device. Inset: EL spectrum and image of the device at 5 V. Copyright 2015, John Wiley & Sons. (c) Coordinates of the devices in CIE space and corresponding EL spectra (insets). Copyright 2021, John Wiley & Sons. (d) Variations of photoluminescence (PL) spectra and PL quantum yields for CBP:*x*% *p*CNQ-TPA films (*x* = 10–100). Copyright 2021, John Wiley & Sons.

Nowadays, ultra-high-definition displays are treated as a key factor to drive the growth of the global 4 K technology market. TADF emitters also contribute to this field. Very recently, an NIR TADF emitter, *p*CNQ-TPA with a quasi-planar structure (Scheme 1), showed great potential for applications in the field of ultra-high-definition displays. The *p*CNQ-TPA-based OLED showed desirable CIE coordinates of (0.69, 0.31) and the record maximum EQE of 30.3%, and is the best red TADF device with Rec. 2020 gamut for UHDTV (as shown in Fig. 2(c)). Besides, by tuning the doping concentration of *p*CNQ-TPA, the NIR device reached an emission peaking at 690 nm and a high PLQY of 90% (Fig. 2(d)). The quasi-planar structure of *p*CNQ-TPA contributed to increasing the light out-coupling ratio to 0.34 for achieving high efficiency of devices.^38^

CNPP was developed as a strong electron-withdrawing group by providing a lower LUMO. CNPP-TPA (Scheme 1) could form intramolecular hydrogen bonds between the introduced nitrogen atoms and adjacent phenyls. The configuration derived from a dihedral angle of 13° effectively enhances structural rigidity to improve the transition probability of the ^1^CT state. According to the data in Tables 1 and 2, CNPP-TPA also became a promising NIR TADF emitter.^39^

Very recently, Wang *et al.* developed a strategy to strengthen the electron-withdrawing capability and reduce the energy gap by attaching three AQ cyano groups. The resulting NIR emitter AQTC-DTPA (Scheme 1) exhibited an emission over 800 nm. The twisted configuration of D and A in AQTC-DTPA inhibited intramolecular electronic communication, but this was made up for by the strong intermolecular border orbital coupling, which was very successful in red-shifting the emission. TADF OLEDs based on AQTC-DTPA showed a record high EQE of 0.51%/0.41%/0.30%/0.23% with the EL peak wavelength at 810/828/852/894 nm, respectively. Increasing the thickness of the light-emitting layer apparently generated a red shift in emission and a higher efficiency of the EL device. For instance, a high EQE of 0.22% was achieved for 40-AQTC-DTPA (40 nm) with the EL peak at 910 nm and radiance of 961 mW Sr^−1^ m^−2^, and an EQE of 0.17% was achieved at 908 nm for 30-AQTC-DTPA (30 nm).^40^

## Pyrazine derivatives with a single donor

Under the same conditions with the electron donor, powerful electron-withdrawing cyanide groups could efficiently minimize the energy gap of molecules and shift the emission wavelength to the NIR region to some extent. Besides, the non-radiative transition was suppressed by the strong intermolecular interactions among cyanide groups, which could induce a rigid microenvironment. The strategy of using intermolecular interactions to build a TADF channel was applied in some classic dicyanopyrazine based materials, such as acenaphtho[1,2-*b*]pyrazine-8,9-dicarbonitrile (AP), acenaphtho[1,2-*b*]quinoxaline-8,9-dicarbonitrile (AQ), acenaphtho[1,2-*b*]pyrazino[2,3-*e*]pyrazine-9,10-dicarbonitrile (AZ) and so on (Scheme 2).

NIR TADF emitters with a single donor also exhibited great properties. Quinoxaline-6,7-dicarbonitrile (QCN) was also a typical acceptor for the NIR TADF emitter. By attaching the triphenylamine group to QCN, the TPA-QCN (Scheme 2) shows sufficient D–A separation and relatively high *Φ*_PL_ with an emission maximum of 733 nm in the neat film.^41^ Phenanthro[4,5-*abc*]phenazine-11,12-dicarbonitrile (PPDCN) has been employed as an NIR TADF acceptor. The rigid planar framework of the PPDCN acceptor showed a beneficial effect on reducing the non-radiative processes and inducing high photoluminescence (PL) efficiency *Φ*_PL_. In addition, C≡N groups could endow the LUMO with a lower energy level which was advantageous for realizing long wavelength emission. TPA-PPDCN (Scheme 2) doped films showed strong deep-red/NIR emission with *Φ*_PL_ of 73–87%. TPA-PPDCN doped devices (Fig. 4(c)) achieved an EQE of 16.4% with NIR emission peaking at 692 nm.^42^

**Fig. 3:**
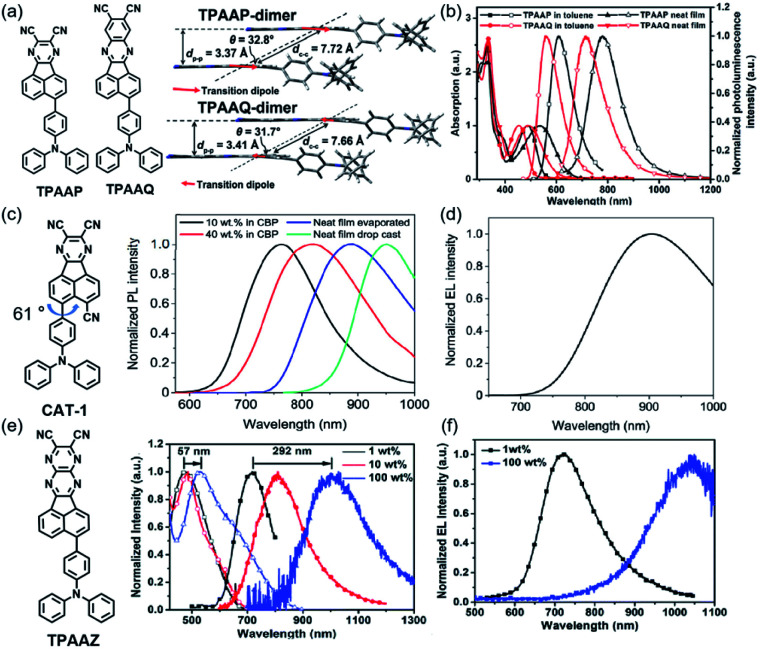
(a) Packing motifs of aggregated dimers of TPAAP and TPAAQ with calculated transition dipoles of S1 denoted as red arrows. (b) Absorption (solid) and PL (hollow) spectra of TPAAP and TPAAQ in toluene and neat solid films. Copyright 2019, John Wiley & Sons. (c) Normalized steady state PL spectra for CAT-1 in doped and neat films. Copyright 2019, American Chemical Society. (d) EL spectrum for an undoped CAT-1 OLED at 5 V. *λ*_max_ = 904 nm. Copyright 2019, American Chemical Society. (e) Absorption (hollow) and emission (solid) spectra of the TPAAZ:CBP doped film and the TPAAZ neat film. Copyright 2020, The Royal Society of Chemistry. (f) Electroluminescence spectra under a voltage of 6 V. Copyright 2020, The Royal Society of Chemistry.

By using the strong electron-donating triphenylamine (TPA), TPAAP (Scheme 2) formed J-aggregates due to its strong intermolecular charge-transfer properties (Fig. 3(a) and (b)). It was confirmed that intermolecular charge transfer could stabilize excited states, reduce the non-radiative decay rate, and further induce highly efficient TADF even in the NIR region according to the experimental and theoretical investigations. In J-aggregates, the splitting of the original singlet exciton states could generate lower-energy and dipole-allowed exciton states with larger transition dipole moments. At the same time, pristine triplet exciton states are almost stable because of the vanishingly small transition dipole moments, thereby resulting in a significant reduction of Δ*E*_ST_.^43^

**Fig. 4:**
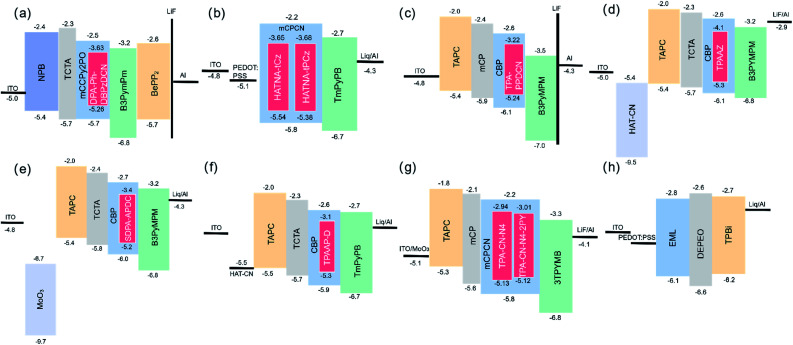
OLED device diagrams. (a) DPA-Ph-DBPzDCN. (Copyright 2018, The Royal Society of Chemistry). (b) HATNA-tCz and HATNA-tPCz. (Copyright 2019, The Royal Society of Chemistry). (c) TPA-PPDCN. (Copyright 2019, American Chemical Society). (d) TPAAZ. (Copyright 2020, The Royal Society of Chemistry). (e) SDPA-APDC. (Copyright 2021, Elsevier). (f) TPAAP-D. (Copyright 2022, Elsevier). (g) TPA–CN–N4 and TPA–CN–N4-PY. (Copyright 2022, The Royal Society of Chemistry). (h) BDC-2. (Copyright 2018, American Chemical Society).

The structure based on AP was further developed. A new NIR TADF emitter, CAT-1 (Scheme 2), with an additional C≡N group provided a low LUMO with a phenyl spacer–acceptor dihedral angle of 61° (Fig. 3(c)), which ensured a narrow calculated Δ*E*_ST_ of 0.11 eV which also greatly enhanced the electron-withdrawing ability. Therefore, the maximum emission wavelength of CAT-1 in the thin-film state was 887 nm. Moreover, the PL emission was further red-shifted with increasing doping ratio. The fabricated NIR device displayed impressive EL with the *λ*_max_ value beyond 900 nm (Fig. 3(d)).^44^

To enlarge the conjugate of the TPAAP moiety, TPAAQ (Scheme 2) with stronger electron-withdrawing ability and high rigidity was investigated as a building block for NIR TADF materials. The neat films of TPAAQ showed NIR emission with a maximum of 716 nm and *Φ*_PL_ values of 16.3 ± 1.6%. For the 1 wt% dopant film in TPBi, the transient PL spectra demonstrated double-exponential decays and the lifetime of delayed fluorescence was 35.7 ms. Through a rational molecular design strategy, J-aggregates with strong ICT character were found in the solid state of TPAAQ. J-aggregates often contain Frenkel excitons and CT excitons which could reduce Δ*E*_ST_ and further enhance TADF by promoting radiative transition. In thin-film states, TPAAQ (doped in TPBi) revealed a remarkable luminescence quantum efficiency of nearly 100%.^43^

Apart from the zygomorphic structured pyrazine derivatives, some asymmetric structures with larger conjugated pyrazine derivatives, such as 11,12-di(pyridin-3-yl)dibenzo[*a*,*c*]phenazine-3-yl(*m*-DPBPZ), were also developed as NIR TADF emitters. Phenoxazine (PXZ) was used as the electron donor to optimize the balance between molecular rigidity and intermolecular stacking. Although *m*-DPBPZ contains rotatable pyridines, which could probably decrease the molecular rigidity, they can suppress molecular π–π packing and thus enhance the emission properties in the non-doped films. As a result, *m*-DPBPZ TADF emitters (Scheme 1) have an extremely small Δ*E*_ST_ of 0.04 eV. At the same time, *m*DPBPZ-PXZ (Scheme 2) showed slightly reduced efficiency with a *Φ*_PL_ of 95 ± 1.3%, and an EQE of 21.7% in the doped OLED. Besides, the EL wavelength of non-doped OLEDs based on *m*-DPBPZ red-shifted to the NIR region with peaks at 680 nm with noteworthy high efficiency with a maximum EQE of 5.2% at corresponding CIE coordinates of (0.68, 0.32).^45^

According to the above studies, aromatic fused rings with an extended conjugation structure have been demonstrated to be a good choice to decrease the band gap into the NIR region. Based on this, DCPA was developed by replacing the naphthalene moiety with the classic anthracene chromophore as the acceptor core. In this way, DCPA could also keep the synergistic electron-withdrawing effect of the cyano group with the adjacent aceanthryleno[1,2-*b*]pyrazine. Asymmetry of the molecular structure was further increased by the introduction of an anthracene core, which contributed to the X-aggregate packing mode in the DCPA-TPA crystal. Moreover, the non-doped devices based on DCPA-TPA and DCPA-BBPA (Scheme 2) showed NIR emission with emission peaks at 838 and 916 nm and maximum EQEs of 0.58% and 0.07%.^46^

Charge-transfer aggregation could significantly stabilize exciton states and reduce the non-radiative decay process ratio; it also induced strong TADF for desirable NIR-II emission from a non-TADF molecule. So, it was regarded as a good strategy to design NIR TADF emitters. TPAAZ (Scheme 2) displayed a gradually red-shifted emission (717 nm to 1010 nm for 1 wt% doped films) in thin-film with the rise of the doping concentration (Fig. 3(c)). The EL spectrum of a non-doped OLED (Fig. 4(d)) is shown in Fig. 3(d).^47^ dpTPAAZ (Scheme 2) with extended conjugation also exhibited remarkable NIR TADF performance. By employing the electron receptor pyrazine on the receptor of dpTPAAP, the emitter not only enhances the ICT process, but also simultaneously makes NIR emission at the single-molecule level come true.^48^ dpTPA, with additional phenyl groups compared to TPA, was considered to beneficially increase the delocalization of the HOMO, which results in red-shifted emission with a high PLQY. In dpTPA, an intermolecular charge-transfer aggregate (CTA) was employed to promote nonadiabatic coupling suppression, which seemed to be a feasible and innovative strategy to realize more high-efficiency NIR emission. The CTA is typically formed by intermolecular CT in the excited state, which ensured excellent photophysical performance and stabilized excited-state energy of the NIR TADF emitter (Fig. 2(c) and (d)). All these factors make the strong TADF performance in dpTPAAP achieve highly efficient NIR EL.

Liao *et al.* developed a spiro-type electron-donating moiety *N*,*N*-diphenyl-9,9′-spirobi[fluorene]-2-amine (SDPA). Benefiting from the strong electron-donating strength of SDPA, SDPA-APDC (Scheme 2) exhibited a narrower bandgap. The highly rigid structure endows SDPA-APDC (Scheme 2) with remarkable thermal stability. The PLQY of SDPA-APDC in the thin film could reach 80.5%. The corresponding EL devices (Fig. 4(e)) exhibited an NIR emission peak at 782 nm with a maximum EQE of 2.55%.^49^

Recently, designing novel NIR TADF molecules by theoretical calculation has become an effective strategy. Lin *et al.* reported several acceptors by changing the position of the cyano group or introducing the phenanthroline into CNBPz. Among them, 44 molecules were selected and studied theoretically. Moreover, the emission spectra of DA-*o*CNPPz, DA-*p*CNBPz, and DA-*p*CNPPz in toluene were simulated in the NIR region. Accordingly, DA-*p*CNPPz (Scheme 2) was demonstrated to exhibit the highest fluorescence efficiency of 20% among reported NIR TADF molecules, with a deep red emission beyond 700 nm in toluene solution.^50^

In 2021, introduction of the cyano group was once again proved to be an effective strategy to rationally regulate the emission wavelengths of TADF emitters to the NIR region (such as CN-TPA, Scheme 2). Devices based on CN-TPA exhibited a leading EQE_max_ of 18.41% at 688 nm and 15.05% at 698 nm.^51^ This work provided new insights on the design of efficient NIR TADF emitters.

By investigating the intrinsic influence of the isotope effect on the luminescence efficiency of TADF emitters, deuterated donors TPA, TPAAP-D and TPAAQ-D (Scheme 2) were developed. As expected, deuteration greatly reduced the non-radiation internal conversion rate, resulting in a significant increase in the PLQY of TADF molecules. Moreover, TPAAP-D achieved an outstanding record with the EL peak at 760 nm (FWHM = 45 nm) and EQE of 2.8%, and the corresponding device structure is shown in Fig. 4(f).^52^ By employing the new electron donor based on tetraphenylethylene (TPE) triphenylamine, CAT-TPE (Scheme 2) realized good solubility and aggregation induced emission behavior. As the first solution-processable NIR TADF molecule based on a fused polycyclic aromatic electron acceptor, TPE-CAT provided a new horizon for NIR TADF emitters.^53^ The combined effects of solvatochromism and aggregation increased the concentration of CAT-TPE in TPBi and the PL could be red-shifted. The PL of a pristine drop-cast film was even further red-shifted close to 1 mm (*λ*_max_ = 961 nm).

Researchers adopt some typical configurations as we mentioned above. Aside from those emitters, a novel D–A_1_–A_2_–A_3_ configuration has also been developed recently. To enhance the electron-withdrawing ability, the acceptor was incorporated by three types of sub-acceptor units. Moreover, according to the experiment results, TPA–CN–N4-2PY (Scheme 2) provided an extended π-backbone, which influenced the EL emission and improved the horizontal ratio of the emitting dipole orientation at the same time.^54^

## Others

**Scheme 3:**
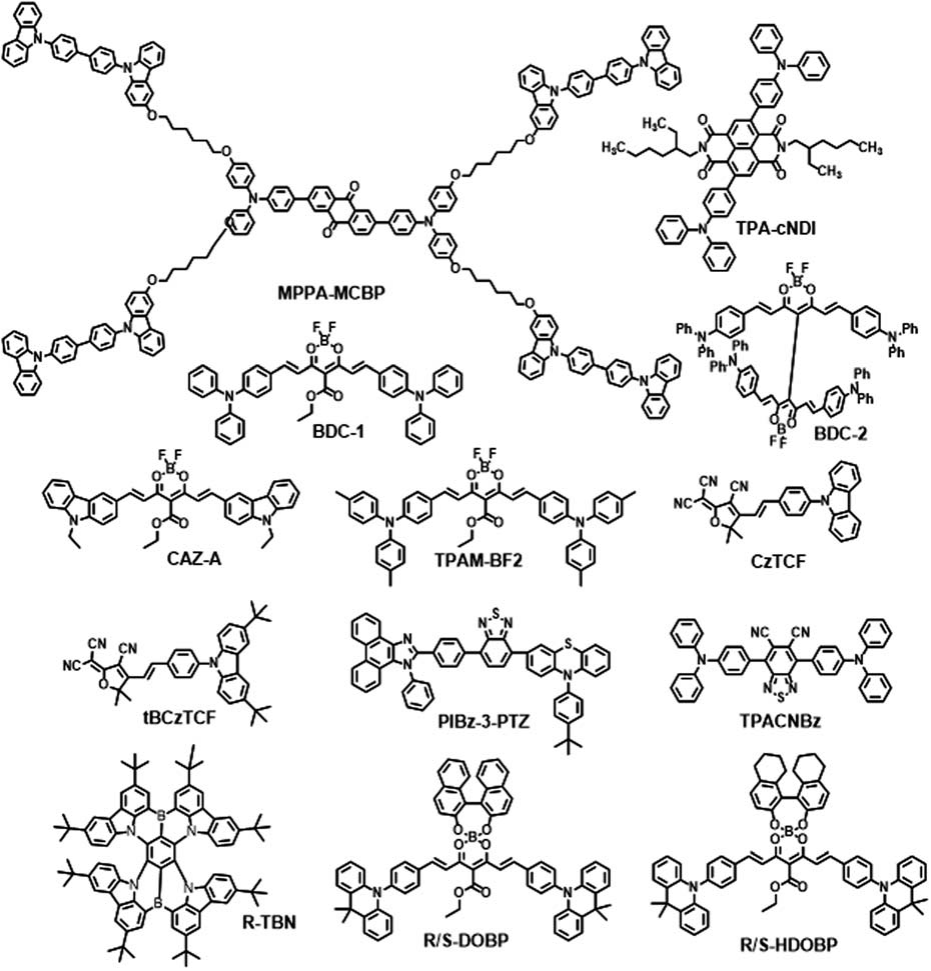
Other types of NIR molecules mentioned in the review.

There are some other excellent electron acceptors without pyrazine also worth discussing due to their outstanding properties as listed in Scheme 3.

Poly(dendrimer)s have provided a new strategy for the development of efficient solution-processed OLEDs in recent years.^57^ Sun and coworkers designed and synthesized a self-host TADF dendrimer for a solution-processed non-doped NIR OLED. In the D–A type dendrimer, the electron donating groups and electron-withdrawing groups combined well with flexible alkyl chains, which can effectively reduce the intermolecular interaction between emitting centers and ensure stable performances in solution-processed OLED devices. It was shown that the spin-coated organic EL device with MPPA-MCBP (Scheme 3) as the NIR emitter featured the highest EQE of 0.62% (emission maximum at 698 nm). This work showed that the design of an autonomous TADF dendrimer with a bipolar tree structure might be a promising strategic solution to improve EL performance.^55^

Data and coworkers developed TPA-cNDI (Scheme 3) as an efficient NIR TADF emitter. Due to the linking phenylene ring being in a non-planar configuration in the solution state, charge transfer processes between TPA and cNDI are promisingly enhanced in a more planar, conjugated geometry in the aggregation state. NIR OLED devices were fabricated based on TPA-cNDI (10 wt% in CBP) with the emission maximum at *ca.* 730 nm and EQE of 2.4%.^56^

Boron difluoride curcuminoid (BDC) derivatives BDC-1 and BDC-2 (as shown in Scheme 3) were composed of one or two triphenylamine (TPA) moieties as the donor group, while the acetylacetonate boron difluoride groups acted as the strong acceptors. Adachi and coworkers reported BDC-1 as an NIR TADF emitter. OLEDs based on BDC-1 showed a maximum EQE of nearly 10%. In addition, NIR amplified spontaneous emission was also detected due to the strong spatial overlap between the hole and electron wavefunctions. The maximum EL emission wavelength could be adjusted from 700 to 780 nm by controlling the polarity of the active medium. With decreasing doping concentration, the PLQY values of the CBP blends increase rapidly, which could reach the highest value of *ca.* 70% with a dopant concentration of 6 wt%.^57^

Ye and coworkers designed and synthesized a solution-processable NIR TADF emitter, BDC-2 (ref. 58) (Scheme 3). NIR OLEDs based on BDC-2 showed a maximum EQE of 5.1% with an emission wavelength of 758 nm. The ASE emission bands were also observed and the maxima could be gradually shifted from 801 to 860 nm at various doping concentrations. BDC-2 in blend films displayed an amplified spontaneous emission band above 800 nm with a threshold as low as 7.5 μJ cm^−2^. These reports illustrated that TADF boron difluoride curcuminoid derivatives were promising candidates for high-performance NIR OLEDs and NIR organic semiconductor lasers. In 2019, Fu *et al.* adopted a TADF organic solid-state laser based on a borondifluoride curcuminoid derivative, CAZ-A (Scheme 3), by employing a Cz derivative as a donor.^59^ Researchers also developed TPAM-BF_2_ by replacing the phenyl groups of BDC-1 with methylphenyl which exhibited better OLED performances.^60^

An acceptor named 2-dicyanomethylene-3-cyano-4,5,5-trimethyl-2,5-dihydrofurance (TCF) with linearly distributed cyano groups was exploited by Xu and coworkers. Based on this acceptor, two NIR TADF materials, named CzTCF and tBCzTCF, were constructed (Scheme 3). The ICT effects and HOMO–LUMO overlaps of these two molecules were significantly improved by the linear distribution of cyano groups in TCF and the styryl π-bridge. All the decay processes of CzTCF and tBCzTCF films exhibited biexponential properties indicating the presence of TADF processes. Moreover, the non-doped solution-processed OLED based on tBCzTCF was successfully fabricated with an emission maximum of 715 nm.^61^

Phenanthro[9,10-*d*]imidazole (PI) has a rigid planar π conjugation and two different unique bonding types of nitrogen atoms which results in high *Φ*_PL_ and thermal stability and ambipolar carrier transport properties. As PI is often used as a weak donor, choosing suitable electron-withdrawing groups could adjust the emission wavelength from the deep-blue to the green range according to previous results.^62^ Lu and coworkers developed three TADF emitters PIBz-10-PTZ, PIBz-10P-PTZ, and PIBz-3-PTZ (Scheme 3) by utilizing PI and PTZ as donors and benzothiadiazole (Bz) as an acceptor. These compounds exhibit different photophysical properties and device performances by changing the linking positions between PTZ and Bz. Among these molecules, PIBz-3-PTZ exhibits a strong DR/NIR emission and aggregation-induced emission properties with a high *Φ*_PL_ of 35% in neat thin films in particular. The non-doped OLEDs achieved a maximum EQE of 2.02% with an emission peak at 672 nm and brightness of up to 3403 cd m^−2^. In addition, the device was able to maintain an EQE of 1.69% at a high luminance of 100 cd m^−2^, with a low roll-off of 16%, suggesting that the non-doped device can keep a relatively high efficiency at very high brightness. These results demonstrate that PI could be a useful donor to construct highly efficient NIR-emission emitters.^63^

A simple and fairly highly electron-deficient receptor, 5,6-dicyano[2,1,3]benzothiadiazole (CNBz), was presented. A typical D–A–D type system was constructed by end-capping with the electron-donating triphenylamine (TPA) unit. TPACNBz (as shown in Scheme 3) exhibited an efficient NIR TADF emission (*λ*_em_ = 750 nm) with a very small Δ*E*_ST_ of 0.06 eV. The device based on TPACNBz exhibited an excellent performance *i.e.*, a high maximum radiance of 10 020 mW Sr^−1^ m^−2^, an impressive maximum EQE of 6.57%, and a peak wavelength of 712 nm.^64^

Recently, TADF materials with polycyclic heteroaromatics embedded in multiple boron (B)- and nitrogen (N)-atoms have also been developed.^65–67^ Introduction of multiple B and N atoms into polycyclic heteroaromatics resulted in the successful formation of restricted π-bonds on the phenyl-core for delocalized excited states, thus narrowing the energy gap. As the first NIR multi-resonance TADF emitter developed in 2021, the R-TNB (Scheme 3) based OLED exhibited an impressive high maximum EQE of 27.6%; the value is the highest recorded among all reported results of NIR TADF devices as summarized in Table 2.^68^ This work provides an effective strategy of multiple B and N atoms in polycyclic heteroaromatics, showing viable potential to generate DR/NIR emitters with bright and efficient emission without nonradiative transitions.

Very recently, two pairs of TADF enantiomers (*R*/*S*-DOBP and *R*/*S*-HDOBP) (Scheme 3) with tetracoordinate boron geometries were developed by Yang *et al. R*/*S*-DOBP and *R*/*S*-HDOBP exhibited high PLQY and efficient reverse intersystem crossing in neat films. The R-DOBP based non-doped solution-processed OLEDs revealed an NIR emission (peaking at 716 nm) with a maximum external quantum efficiency of 1.9% and high exciton utilization efficiency of 86%, and are some of the best solution-processed non-doped NIR-OLEDs.^69^

**Scheme 4:**
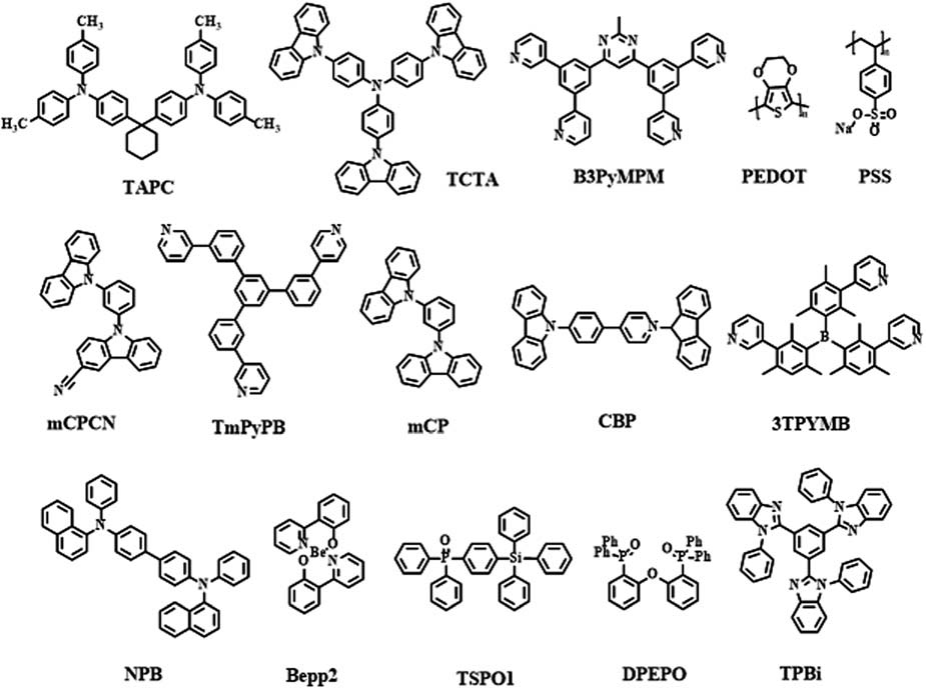
Host materials mentioned in the review.

**Fig. 5:**
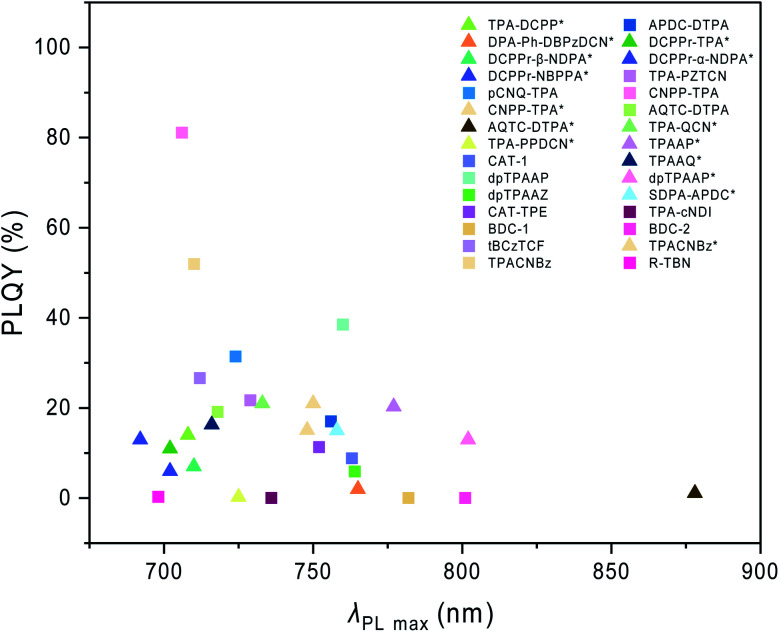
PLQY summary of the representative NIR TADF emitters. * Data were acquired from the neat film.

**Fig. 6:**
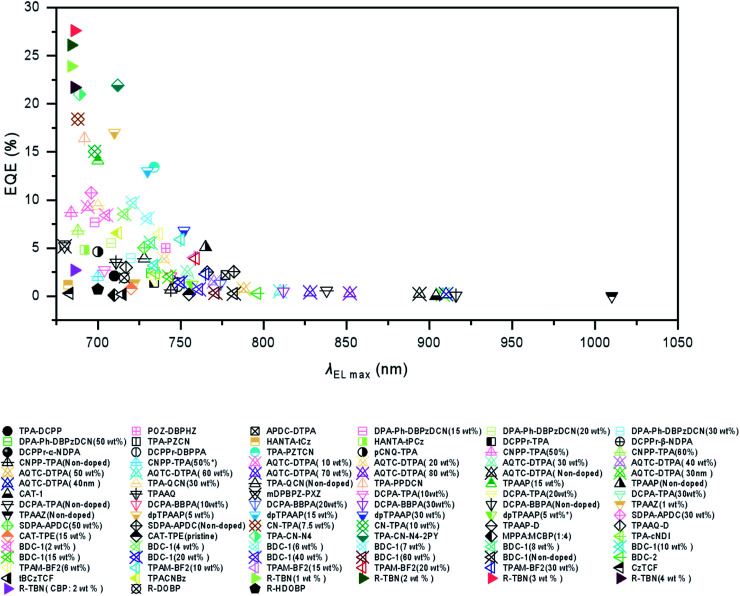
EQE summary of the representative NIR TADF OLEDs.

In this review, besides Schemes 1–3 as we mentioned above, we also listed the host materials mentioned in the review in Scheme 4. Moreover, the photophysical properties of TADF emitters we have mentioned before can be found in Table 1, and the corresponding device structures and performances are further listed in Table 2. The chemical structures mentioned in Table 2 are also shown in Scheme 4 to make them clearer. Besides, we also summarize the PLQYs of the representative NIR TADF emitters in Fig. 5 and EQEs of the representative NIR TADF OLEDs in Fig. 6. In Fig. 5, an asterisk (*) indicates that the data for that emitter were acquired from the neat film, while for the other emitters not marked by an asterisk they were acquired from the doped film. They are also shown in the patterns, where triangles represent data from neat films and squares represent data from doped films. In Fig. 6, the same emitter-based OLEDs are shown in the same pattern but different colors to make it easier to read. With the supplement of Fig. 5 and 6, we believe that our review can further provide inspiration and ideas for readers to design NIR TADF materials with better performance by intuitively summarizing the performance of the emitter and device.

(TAPC: 1,1-bis[4-[*N*,*N*-di(*p*-tolyl)-amino]-phenyl]cyclohexane, TCTA: 4,4′,4′′-tris(carbazol-9-yl)-triphenylamine, B3PymPm: 4,6-bis(3,5-di(pyridin-3-yl)phenyl)-2-methylpyrimidine, ITO: indium tin oxide, PEDOT: poly(3,4-ethylenedioxythiophene), PSS: poly(styrenesulfonic acid), mCPCN: 9-(3-(9*H*-carbazol-9-yl)-phenyl)-9*H*-carbazole-3-carbonitrile, TmPyPB: 1,3,5-tri(*m*-pyrid-3-yl-phenyl)benzene, mCP: *N*,*N*-dicarbazolyl-3,5-benzene, CBP: 4,4′-di(9*H*-carbazol-9-yl)-1,1′-biphenyl, 3TPYMB: tris-[3-(3-pyridyl)mesityl]borane, NPB: *N*,*N*′-bis(naphthalen-1-yl)-*N*,*N*′-bis(phenyl)-benzidine, Bepp2: bis[2-(2-hydroxyphenyl)-pyridine]beryllium, TSPO1: diphenyl[4-(triphenylsilyl)phenyl]phosphine oxide, DPEPO: bis[2-(diphenylphosphino)phenyl]ether oxide, TPBi: 2,2′,2′′-(1,3,5-benzinetriyl)-tris(1-phenyl-1-*H*-benzimidazole)).

## Conclusion and outlook

In this review, we have summarized the recent progress in NIR TADF emitters according to their electron-withdrawing moieties and their potential applications in the field of OLEDs. To clarify the specific molecular design strategy, we divided all of them by electron acceptors and listed them in Schemes 1–3, respectively. It was significant to reveal that suitable electron-withdrawing groups for NIR TADF emitters often had rigid structures and torsion angles with donors to ensure a small Δ*E*_ST_ and suppress the loss of non-radiative energy. As we have classified, pyrazine derivatives play an important role in NIR TADF emitters; nowadays, multi-resonance emitters like multiple B- and N-atom embedded polycyclic heteroaromatic molecules and so on also exhibited great potential in the fields of NIR TADF emitters. The key factor for developing decent NIR TADF emitters is choosing suitable electron-donating moieties and electron-withdrawing moieties. Although many NIR TADF emitters have been developed, few of the above NIR OLEDs could achieve maximum EQE above 10%. Geometry configuration, intermolecular interactions, substituents, heteroatoms/heterocycles, *etc.* could be crucial factors that need to be considered when designing the new NIR TADF molecular structure of the materials.

According to this review, this research field has some impressive progress but is still in its infancy years. Future research and development is likely to move forward on two fronts in the study of NIR TADF emitters and OLEDs: organic NIR TADF emitters including conjugated and donor–acceptor charge transfer compounds and polymers. Applications will always be influenced by the chemical, physical, optical, and processability features. For D–A type organic NIR TADF emitters, based on the fact that triphenylamine derivatives were used for most donors, it could be foreseeable that the investigation of novel electron-withdrawing groups for new types of receptors will be prosperously developed in the future. Moreover, for scholarly study and real-world use, more structural receptor types ought to be developed. Therefore, more receptor developments deserve attention especially multiple boron (B)- and nitrogen (N)-atom embedded polycyclic heteroaromatic molecules and so on with extended conjugation and small Δ*E*_ST_. As a result, more useful molecular design strategies would be adopted. Considering that polymer dots could be used as powerful probes for bioimaging,^70^ biosensing,^71^ and photodynamic therapy,^72^ NIR TADF polymers with higher efficiencies and stronger penetration will be more worthy of exploration with more excellent practical applications.

In terms of the future development of OLEDs, we believe more solution-processed NIR OLEDs would be designed and developed based on the TADF NIR emitters, thus significantly reducing production cost. Production of commercially available flexible OLEDs is now based on low temperature polysilicon. With the development of technology, the excellent properties of TADF materials will play a role since they can not only be exposed to high temperature but also emit better emission intensity at high temperature. At the same time, the luminescence efficiency and lifespan will have greater development potential in the future. More applications like all-organic optical upconversion devices would prosper based on the development of NIR TADF emitters. The investigation of NIR TADF emitters and related OLED devices will witness rapid growth in coming years.

## Author contributions

Yuxin Xiao, Hailan Wang, and Zongliang Xie made equal contributions to this review. All the authors were involved in the revision of this manuscript.

## Conflicts of interest

The authors declare that they have no known competing financial interests or personal relationships that could have appeared to influence the work reported in this paper.

## Supplementary Material
